# Engineering Ruthenium-Based Electrocatalysts for Effective Hydrogen Evolution Reaction

**DOI:** 10.1007/s40820-021-00679-3

**Published:** 2021-07-24

**Authors:** Yingjie Yang, Yanhui Yu, Jing Li, Qingrong Chen, Yanlian Du, Peng Rao, Ruisong Li, Chunman Jia, Zhenye Kang, Peilin Deng, Yijun Shen, Xinlong Tian

**Affiliations:** State Key Laboratory of Marine Resource Utilization in South China Sea, Hainan Provincial Key Lab of Fine Chemistry, School of Chemical Engineering and Technology, Hainan University, Haikou, 570228 People’s Republic of China

**Keywords:** Hydrogen evolution reaction, Ruthenium-based catalysts, Performance, Electrochemical water splitting

## Abstract

The investigation of highly effective, durable, and cost-effective electrocatalysts for the hydrogen evolution reaction (HER) is a prerequisite for the upcoming hydrogen energy society. To establish a new hydrogen energy system and gradually replace the traditional fossil-based energy, electrochemical water-splitting is considered the most promising, environmentally friendly, and efficient way to produce pure hydrogen. Compared with the commonly used platinum (Pt)-based catalysts, ruthenium (Ru) is expected to be a good alternative because of its similar hydrogen bonding energy, lower water decomposition barrier, and considerably lower price. Analyzing and revealing the HER mechanisms, as well as identifying a rational design of Ru-based HER catalysts with desirable activity and stability is indispensable. In this review, the research progress on HER electrocatalysts and the relevant describing parameters for HER performance are briefly introduced. Moreover, four major strategies to improve the performance of Ru-based electrocatalysts, including electronic effect modulation, support engineering, structure design, and maximum utilization (single atom) are discussed. Finally, the challenges, solutions and prospects are highlighted to prompt the practical applications of Ru-based electrocatalysts for HER.

## Highlights

- Four main strategies for improving the hydrogen evolution reaction (HER) performance of Ru-based catalysts were summarized.
- The source of HER activity of Ru-based catalysts is discussed in terms of catalytic mechanism.
- The current states, challenges and prospects were specifically provided for Ru-based catalysts.

## Graphical abstract

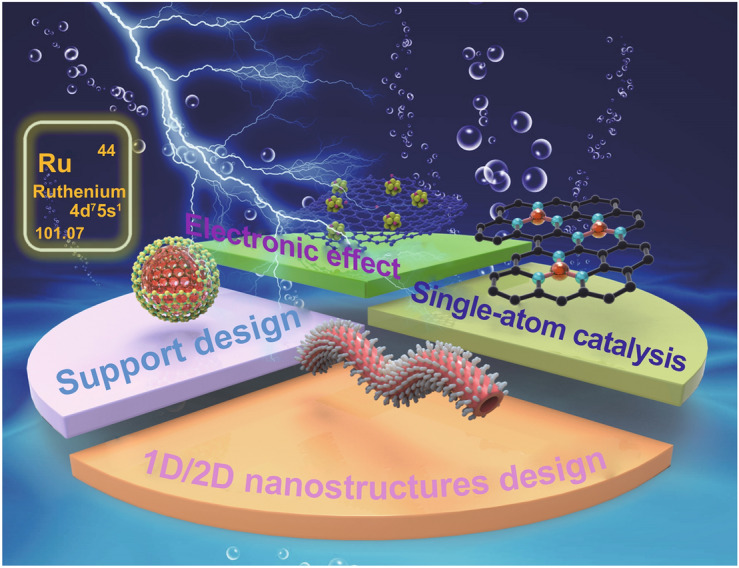

## Introduction

Currently, the lack of traditional primary energy (oil, coal, and natural gas) necessitates a solution to the current energy crisis; thus, ultra-high energy density (146 kJ g^−1^) hydrogen (H_2_) that is clean and environmentally friendly has captured people’s attention in this regard [1]. H_2_ as a fuel only produces water, compared to conventional fossil fuels that release carbon dioxide, nitric oxide, and sulfides. With the rapid development of hydrogen fuel cells, hydrogen energy is expected to solve a series of environmental problems, such as global warming and air pollution. However, the main H_2_ production methods currently used in industry are steam reforming and coal gasification, and they are still based on fossil fuel production and are uneconomical and energy-consuming [2, 3]. Alternatively, electrochemical water splitting is a desirable and economical way to produce hydrogen with high efficiency, high H_2_ purity (> 99%), and abundant reactants (water resources) [4]. However, the reaction rate of the hydrogen evolution reaction (HER) on the cathode is slow, and an efficient and stable catalyst is urgently required to accelerate the reaction rate.

Platinum (Pt) is the most effective HER catalyst, while its wide application is limited due to its high cost and low reserves [5–8]. In the past few decades, several non-noble metal catalysts or metal-free catalysts have been developed for the HER, but their poor activity and stability still cannot meet the requirements to replace Pt-based catalysts [9–13]. Owing to the similar hydrogen bond strength (~ 65 kcal mol^−1^) between ruthenium (Ru) and Pt, Ru is considered a promising HER catalyst [14]. Notably, the price of Ru has great advantages over many precious metals, such as Pt (1131 $ per oz), Pd (2335 $ per oz), Rh (29,185 $ per oz), or Ir (5075 $ per oz), and the price of Ru is only 1/4 of Pt. Therefore, Ru-based HER catalysts have been widely investigated and are expected to replace Pt-based HER catalysts.

Although Ru-based HER catalysts show great potential, their research and industrial applications are still in infancy, and there is still room for improvement and unlimited potential in the HER performance of Ru-based catalysts. There are some review articles on Ru-based catalysts for HER in recent years [1, 14–20], and they mainly provide a detailed synthesize processes of the catalysts [16, 17, 19, 20], or they provide a summary of the performance optimization design via part of four major strategies discussed in this article, that is, electronic effect modulation, support engineering, structure design, and maximum utilization [1, 14, 15, 18]. At present, more incisive summary is still needed to improve the understanding of the performance optimization of Ru-based HER catalysts, and we need to deeply understand the HER mechanism and metal characteristics for developing advanced Ru-based electrocatalysts, and make according optimizations. Therefore, summarizing the current strategies to improve the HER catalytic performance and find an effective path to further boost the HER performance of Ru-based catalysts is necessary. This review summarizes the progress of Ru-based catalysts for the HER in recent years and introduces its current applications. First, the basic principles of the HER, some descriptors of the computational activity, and electrochemical activity are briefly introduced. Subsequently, the typical design methods that enhance the HER activity of Ru-based catalysts are analyzed, including the electronic effect regulation, support and structure engineering, and maximum utilization (single atom). Finally, the challenges and prospects of Ru-based catalysts for the HER are proposed.

## Basic Principles of the HER

### Mechanisms of the HER

The HER occurs at the cathode in an electrolytic cell. As the protons are provided in different ways in acidic and alkaline media, the process of HER is also different. Under acidic conditions, the first step is the electrochemical adsorption step, i.e., the Volmer step. The H^+^ in the solution obtains electrons and adsorbs onto the surface of the material to form a hydrogen intermediate (H*) that is the basic prerequisite for H_2_ evolution. Subsequently, two competitive reactions of H_2_ evolution occur: one is the electrochemical desorption step, namely the Heyrovsky step, and H* combines a H^+^ and an electron in the solution to generate H_2_ for removal; the second is the chemical desorption step (Tafel step), and two adjacent H* produced by the Volmer step are reorganized directly to form H_2_ on the catalyst surface [21]. Under alkaline or neutral conditions, H* is obtained through water splitting, which is also divided into three elementary reactions. The specific reactions are presented in Table 1. Therefore, by calculating the Tafel slope, the rate-determining step (RDS) of the reaction can be evaluated as either the Volmer–Heyrovsky or Volmer–Tafel path. Notably, additional energy is required to obtain H* under alkaline conditions, resulting in a slower kinetic rate for the alkaline HER. Considering Pt as an example, in alkaline medium, owing to the high energy barrier of water splitting, the reaction rate is often two to three orders of magnitude slower than that in acidic media [22]. However, the issue of stability and corrosion of both catalysts and reaction devices is a major challenge in acidic conditions; thus, improving the hydrolysis kinetics in alkaline media has become the primary direction of current research.

**Table 1:** Possible reaction pathways of HER under acidic, alkaline and neutral conditions

Step	Tafel slope (25 ℃)	Condition	Pathway
Volmer	$b = \frac{{2.3RT}}{{\alpha F}} \approx 120~{\text{mV~dec}}^{{{{ - 1}}}}$	Acidic Alkaline and neutral	${\text{H}}^{ + } + {\text{e}}^{ - } \to {\text{H}}^{*}$ ${\text{H}}_{2} {\text{O}} + {\text{e}}^{ - } \to {\text{H}}^{{\text{*}}} + {\text{OH}}^{ - }$
Heyrovsky	$b = \frac{{2.3RT}}{{\left( {1 + \alpha } \right)F}} \approx 40~{\text{mV~dec}}^{{{{ - 1}}}}$	Acidic Alkaline and neutral	${\text{H}}^{*} + {\text{H}}^{ + } + {\text{e}}^{ - } \to ~{\text{H}}^{*}$ ${\text{H}}^{*} + {\text{H}}_{2} {\text{O}} + {\text{e}}^{ - } \to ~{\text{H}}_{2} + {\text{OH}}^{ - }$
Tafel	$b = \frac{{2.3RT}}{{2F}} \approx 3{\text{0~mV~dec}}^{{{{ - 1}}}}$	Acidic Alkaline and neutral	${\text{H}}^{*} + {\text{H}}^{*} \to ~{\text{H}}_{2}$ ${\text{H}}^{*} + {\text{H}}^{*} \to ~{\text{H}}_{2}$

*R*, Ideal gas constant; *T*, Kelvin temperature; *α*, Symmetrical coefficient, 0.5; *F*, Faraday constant

### Descriptors of Computational Parameters

**Fig. 1:**
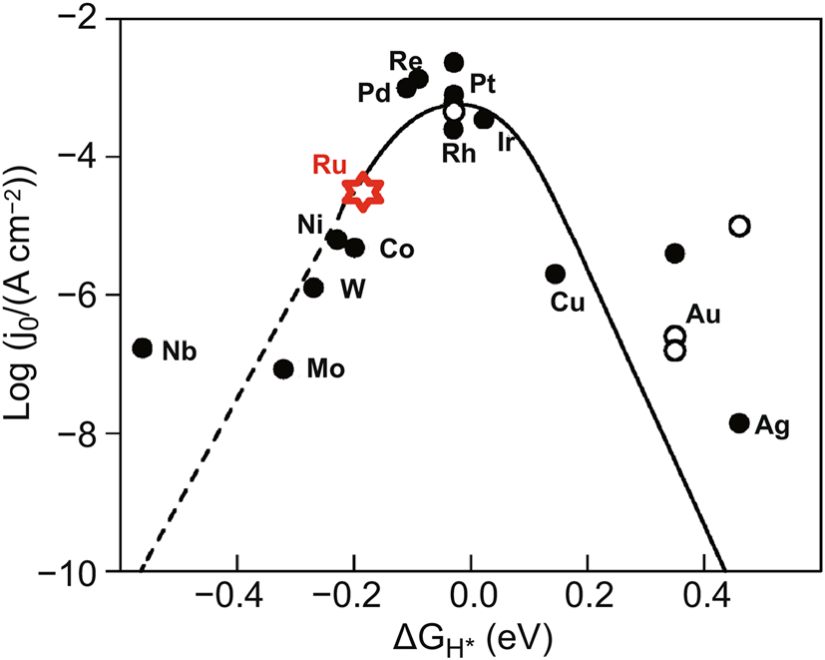
Volcano plot of hydrogen adsorption free energy (ΔG_H*_) of different metal catalysts in HER. With the exception of Ru, the data for the other metals are from the summary of Nørskov et al. High H coverage of metals on the left side of the volcano (1 monolayer (ML)), lower on the right side (0.25 ML). The dashed line indicates that the metals which bind H stronger than 0.2 eV H^−1^ usually form oxides at U = 0 V. The open circles are (111) data, whereas the filled circles are polycrystalline [25]. The data of Ru are from Hoster et al., corresponding to the calculation data of Ru (0001) at 1.1 ML [24]. With permission from the American Chemical Society and Springer Nature

The formation and desorption processes of H* determine the mechanism and rate of HER, and the adsorption and desorption strength of H* on the catalyst surface can be evaluated by calculating the free energy of hydrogen adsorption (Δ*G*_H*_) using density functional theory (DFT) calculations [23]. According to the Sabatier principle, an appropriate interaction between the catalyst and reactant is vital in improving the catalytic rate. The high Δ*G*_H*_ value indicates that the adsorption of H* is weak, implying that it is difficult for H* generation; however, a low Δ*G*_H*_ value indicates that the adsorption of H* is strong, resulting in the difficulty of H_2_ desorption. Therefore, the catalyst exhibited an excellent HER performance, with Δ*G*_H*_ ≈ 0. The volcano plot, which is established with the Δ*G*_H*_ and exchanges current density (j_0_), can intuitively obtain the order of the adsorption strength of each metal for H* (Fig. 1) [24, 25]. The catalyst at the top of the volcano (i.e., Δ*G*_H*_ ≈ 0 and the largest j_0_) shows the best HER performance. Additionally, under alkaline conditions, the kinetic barrier of water dissociation (*E*_b_) and the free energy of hydroxyl desorption (Δ*G*_OH*_) are also indicators of the HER activity.

### Descriptors of the Electrochemical Activity

The general electrochemical parameters for HER are the overpotential, Tafel slope, exchange current density (*j*_0_), turnover frequency (TOF), electrochemical surface area (ECSA), electrochemical impedance spectrum (EIS), Faraday efficiency (FE), and stability. (1) Overpotential: the theoretical driving thermodynamic voltage of the HER is 0 V (vs. RHE), while additional voltage is often required to drive the reaction because of the activation barriers and resistance in the electrochemical systems, which is called the overpotential (η). The current density of 10 mA cm^−2^ is equivalent to 12.3% efficiency of a solar water-splitting device [26]. Therefore, the overpotential at a current density of 10 mA cm^− 2^ (*η*_10_) is generally used as the comparison standard for different catalysts. (2) The Tafel slope, an inherent property of the catalyst, is determined by the RDS. The reaction path can be determined based on the Tafel slope, which is vital in explaining the catalyst mechanism of the reaction. The Tafel slope can be calculated by *η* = b log (*j*/*j*_0_), where b is the Tafel slope, and j and j_0_ are current and exchange current densities, respectively. (3) TOF is a vital parameter for evaluating the intrinsic activity of catalysts, which represents the number of H_2_ moles per unit time produced at each catalytic site at a given potential. As the number of active sites is difficult to determine, the TOF calculation is not accurate. Therefore, Ma et al. [27] described a method for the precision testing of TOF. (4) ECSA can be used to represent the effective catalytic area of the catalyst, which is often measured by the double-layer capacitance (*C*_dl_) [28] or underpotential deposition (UPD) [29]. For Pt-based materials, the ECSA can also be obtained using the coulombic charges integrated under the cyclic voltammetry (CV) curves of hydrogen adsorption [30]. However, accurate measurement is not easy; thus, the ECSA is often used for the comparison of similar component materials. (5) EIS can obtain information about each interface in the catalytic system. The charge transfer resistance (*R*_ct_) can also be obtained by fitting the diameter of the semicircle in the high-frequency region. A small *R*_ct_ indicates a high charge transfer efficiency and fast reaction rate. (6) FE describes the ratio of experimental to theoretical hydrogen production. The theoretical hydrogen production can be obtained by integrating the current–time (*i–t*) curve, and the experimental hydrogen production can be measured by the drainage method or gas chromatography. The closer the FE is to 100%, the higher is the catalytic selectivity. (7) Stability is a significant indicator for evaluating the service life of catalysts, which is often measured by the chronoamperometry/chronopotentiometry (*i–t/p–t*) method or CV.

## Electronic Effect Modulation

The HER efficiency of Ru-based catalysts can be improved by accelerating the water dissociation, reducing the hydrogen adsorption and the reaction barriers, and changing the electronic structure of Ru atoms by heteroatom doping. The high electronegativity of Ru (Pauling scale = 2.2) leads to a strong adsorption capacity for protons, resulting in the unfavorable desorption of H on the Ru surface, and it is known that Ru exhibits a more negative ΔGH* compared to Pt from the volcano plot [24]. Therefore, the electronegativity of Ru can be reduced by introducing heteroatoms that take away the electrons from Ru nucleus, increasing the desorption ability of Ru to hydrogen, thus enhancing the reaction rate of Heyrovsky or Tafel step [31, 32]. It is worth noting that the heteroatom only plays a regulatory role and does not act as the active site, while Ru is still regarded as the active center of HER. In addition to enhancing the rate of hydrogen desorption, regulating the rate of the Volmer step by accelerating the dissociation of water is also an effective pathway to improve the HER performance. The HER activity of Ru can be enhanced by a doping strategy that accelerates the nucleophilic attack of water on the active center Ru and weakens the H–OH bond [28, 33]. When the introduced species is sufficiently strong for water decomposition, the doped species at this point is likely to become the active center for water dissociation, and the role of Ru acts as the active center for hydrogen evolution. Recent studies have focused on the impact of anion doping on the hydrogen evolution activity of Ru through simple electronic effects. Additionally, it is a promising method to improve the HER activity of Ru-based catalysts by regulating the second metal active sites and strengthening the electronic interaction between the active sites and carriers. Recently, several new Ru-based HER catalysts have been developed through doping strategies, showing excellent electrocatalytic performance.

### Non-metal Doping

**Fig. 2:**
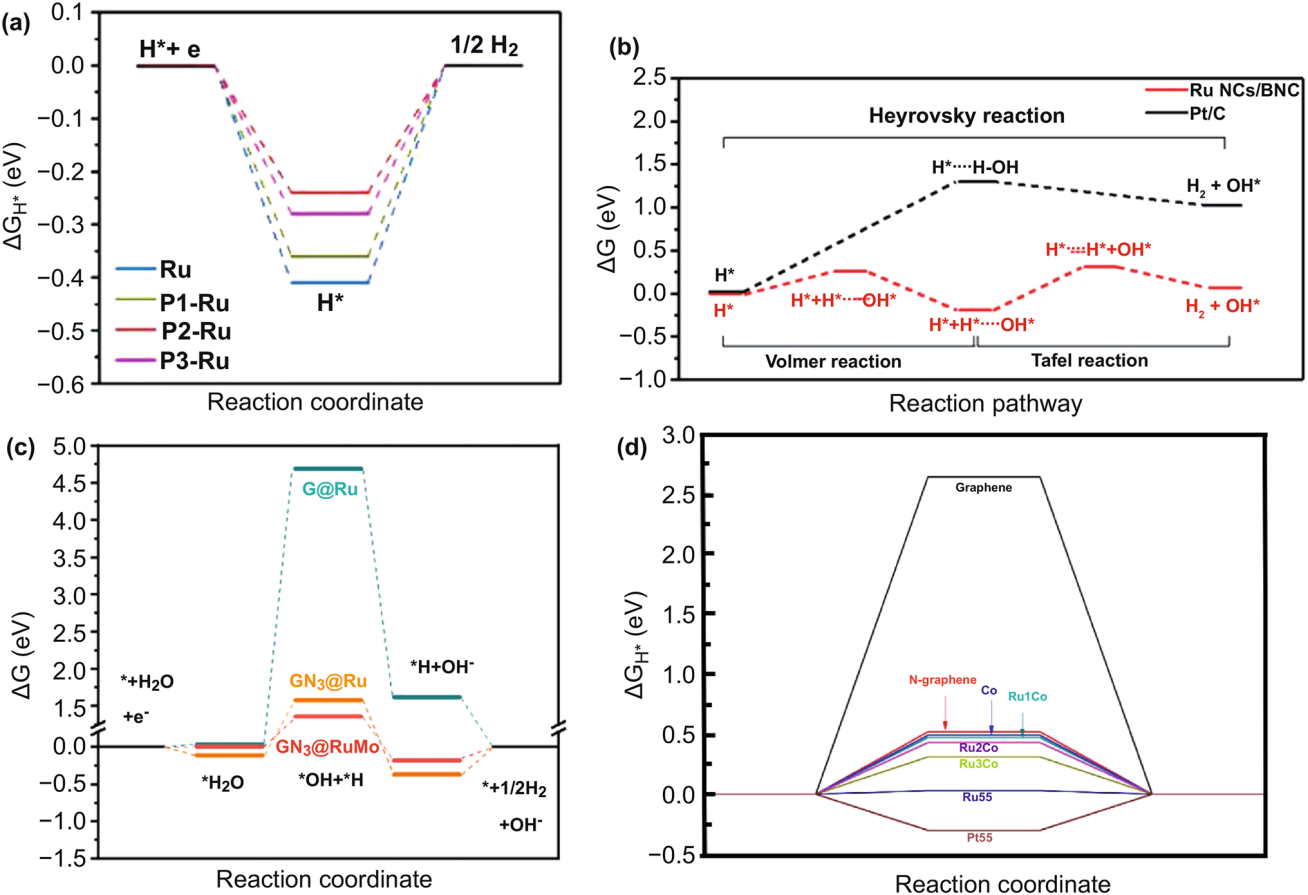
Effect of different doping strategies on free energy of reaction intermediates: **a** ΔG_H*_ on Ru, P1-Ru, P2-Ru, and P3-Ru [31], with permission from the American Chemical Society. **b** Initial, intermediate, and final transition state free energy of Ru NCs/BNC and Pt/C in HER [33], with permission from the Elsevier Ltd. **c** Free energy of each reaction stage of RuMo alloying [47], with permission from the Wiley–VCH Verlag GmbH & Co. KGaA, Weinheim. **d** ΔG_H*_ value under Co-doped Ru-based catalyst [41], with permission from the Nature Publishing Group

The H desorption behavior of Ru is weaker than that of Pt [14, 34, 35], thus regulating the desorption capacity of H on Ru is proven to be effective for improving the HER efficiency of Ru-based catalysts. Luo et al. [31] developed P-doped Ru supported on XC-72 carbon (P-Ru/C) as a highly efficient HER electrocatalyst in alkaline media. P-Ru nanoparticles (NPs) were synthesized via colloidal synthesis, and the P content was controlled by different pyrolysis temperatures and times. The resultant P-Ru/C exhibited an overpotential of 31 mV to reach a current density of 10 mA cm^−2^ in 1 M KOH, which is considerably lower than that of Pt/C (39 mV) and Ru/C (103 mV). The Tafel slope, which is 105 mV dec^−1^ for P-Ru/C, is smaller than those of Pt/C (114 mV dec^−1^) and Ru/C (129 mV dec^−1^). This also indicates that the reaction kinetics were determined by the electrochemical adsorption. Through mass normalization, P-Ru/C (1.03 mA μg^−1^ at *η* = 50 mV) also showed twice as much mass activity as Pt/C (0.45 mA μg^−1^). Furthermore, the catalyst exhibited superior HER stability after the CV testing. To further determine the active site of the catalyst and the contribution of P doping to the HER in alkaline media, density functional theory (DFT) calculations was performed (Fig. 2a). The results showed that the electronic structure of Ru atoms changed with the doping of P, and P obtained electrons from Ru. As the positive charge in Ru atoms increased, the adsorption of H on P2-Ru weakened, and this promoted the desorption of H_2_ and increased the HER efficiency.

Under alkaline conditions, the acquisition of H protons depends on the dissociation of H_2_O; thus, reducing the energy barrier of the H–OH bond is also crucial for improving the HER efficiency. Sun et al. [33] prepared Ru nanoclusters (NCs) anchored on B-/N-doped graphene (BNG) (Ru NCs/BNG). Boron doping into graphene during pyrolysis promotes the formation of ultrafine Ru NCs with a diameter of 0.5–1 nm. The electrocatalytic activities of Ru NCs/BNG were compared with those of Ru NPs/NG and commercial 20 wt% Pt/C in 1.0 M KOH. The Ru NCs/BNG showed outstanding electrocatalytic performance, such as small overpotential at 10 and 50 mA cm^−2^ (14 and 50 mV, respectively), superior durability in both electrolytes, and a low Tafel slope (28.9 mV dec^−1^). According to the Tafel slope, the rate-determining equation (RDE) of Pt/C is the Heyrovsky reaction, and that of Ru NCs/BNG is the Tafel reaction. These performances are comparable to, or even better than, those of Pt/C and Ru NPs/NG for the HER in alkaline solutions. The doping of B reduced the electronegativity of Ru, promoted the nucleophilic attack of H_2_O, and accelerated the fracture of the H–OH bond, and thus promoted the hydrolysis and dissociation of the HER under alkaline conditions. To understand the high electrocatalytic activity after the B doping, DFT was employed to calculate the activation energies of the transformation of H_2_O to H_2_ in Ru NCs/BNG and Pt/C (Fig. 2b). For Pt/C, the Heyrovsky reaction energy barrier reached 1.3 eV (125.9 kJ mol^−1^) and the Volmer reaction energy barrier of Ru NCs/BNG was 0.26 eV (25.2 kJ mol^−1^). This indicates that the doping of B optimizes the electronic structure of Ru and promotes the dissociation of water. Additionally, the doping of S can weaken the H–OH bond and promote water splitting [28].

Doping with various non-metal elements can also improve the HER activity of Ru-based catalysts through the coordination effect. Zhou et al. [36] reported ultrafine S-doped RuP nanoparticles being homogeneously embedded in an N-, P-, and S-codoped carbon sheet (S-RuP@NPSC) by pyrolysis. The mass activity was 22.88 times that of Pt/C at an ultra-low loading of 0.8 wt%. Theoretical calculations confirmed that the surface Ru (− 0.18 eV) and P (0.05 eV) atoms were the HER catalytic active sites in 1 M KOH. Doping with heteroatoms (N, P, S) improves the conductivity of the carrier and modulates the electron distribution around Ru by S and P atom doping to induce a synergistically enhanced reactivity toward the HER.

### Transition Metal Doping

Previous studies have shown that the charge distributions and surface properties of Ru can be optimized by alloying transition metal atoms such as Co and Ni into the Ru lattice, thereby improving its catalytic activity [37–40]. Su et al. [41] developed a highly efficient and stable electrocatalyst composed of Ru and Co bimetallic nanoalloy encapsulated in nitrogen-doped graphene layers (RuCo@NC). The catalysts showed a low overpotential of only 28 mV at 10 mA cm^−2^, and the activity was maintained well after 10,000 cycles of durability testing in alkaline solution. DFT calculations showed that the electron transfer rate from the Co core to the outer graphene increased with the introduction of Ru atoms (Fig. 2d), and the C–H bond energy was strengthened in the reaction process, thereby reducing the ΔG_H*_ of the HER of graphene. Lu et al. [32] reported a hybrid material, Ru-M (M = Ni, Mn, Cu) bimetallic nanoparticles and carbon quantum dots (RuM/CQDs), for the efficient HER. During synthesis, the abundant functional groups (–COOH and –OH) on the surfaces of the CQDs can coordinate with the RuM ions with empty d orbitals to form a relatively stable CQDs-Ru ion coordination composite. The RuM NPs are restricted between the CQDs to form ultrafine nanocrystals with stable structures that effectively prevent the agglomeration and growth of the NPs during the reaction [42, 43]. The catalyst has excellent HER performance at different pH. In particular, the RuNi/CQDs show small overpotential at 10 mA cm^−2^ (13 mV at 1 M KOH, 58 mV at 0.5 M H_2_SO_4_, and 18 mV at 1 M PBS) and long-term durability in acidic, neutral, and alkaline media after 10,000 cycles. As Ni was doped into the lattice of Ru, the d-band center of Ni 3d is downshifted by 1.25 and 1.27 eV for hcp RuNi surfaces, resulting in a more electron-rich state for Ni and electron deficiency for Ru. This behavior not only stabilizes the RuNi particles but also reduces the over-binding effect of H on Ru surfaces, which improves the HER activity.

Heterostructures showed great potential in the field of catalytic energy conversion because of the fascinating synergism of different components in tuning electronic structures for promoted surface catalysis, and the interface charge distribution can also be realized by adjusting different components [44–46]. Zhuang et al. [47] devised a facile and scalable fabrication of a novel heterostructure RuMo nanoalloy-embedded 2D porous carbon (2DPC-RuMo) nanosheet with hard-templating synthesis and anion-exchange processes. The unique structures of the 2DPC-RuMo nanosheets obtained by alloying Mo atoms into the Ru lattice led to an excellent electrocatalytic HER activity with an extremely low overpotential (18 mV at 10 mA cm^−2^ in1 M KOH), an ultrasmall Tafel slope (25 mV dec^−1^), and a high turnover frequency (TOF) of 3.57 H_2_ s^−1^ at 50 mV. Theoretical calculations shows that optimizing the doping of Mo and N (Fig. 2c) promoted the charge redistribution on the Ru surface, and the energy barrier of H-adsorption and the free energy of the reaction pathway are reduced, thus effectively improving the HER efficiency.

Copper (Cu) has been applied in many catalytic systems due to its excellent electrical conductivity. Experimental and theoretical calculations have confirmed that the introduction of Cu in HER can regulate the electronic structure of Ru-based catalysts, thus the catalyst shows optimal H desorption capacity [48]. Cheng et al. [49] reported a facile wet chemistry method for in situ growth of amorphous RuCu nanosheets on crystalline Cu nanotubes (3D RuCu NCs). The obtained catalyst only needs 18 and 73 mV to deliver the current density of 10 mA cm^−2^ for HER in alkaline and neutral media, respectively. It’s proposed that the excellent HER performance comes from the amorphous phase with many unsaturated bonds between Ru and Cu atoms, facilitating the adsorption of reactants. In addition, the crystalline Cu with superior conductivity can promote the transfer of electrons. Furthermore, the electrocatalytic performance of the catalyst exhibits almost no attenuation after 30 h durability test due to its unique 3D space structure effectively prevented the aggregation of nanosheets.

Therefore, the intrinsic disadvantages of Ru-based catalysts for HER can be compensated by the electronic effect modulation, which makes its performance similar or even superior to Pt-based catalysts. Based on the above discussion, we believe that the main purpose of electronic effect modulation is to optimize the adsorption and desorption behavior of Ru-based catalysts for various reaction intermediates in the HER process, and the doping elements should be reasonably selected according to the catalytic application environments. The properties of Ru-based HER catalysts doped with different elements in recent years are presented in Table 2.

**Table 2:** HER performance doped with different elements at 1.0 M KOH

Catalysts	Doped elements	Loading (mg cm^−2^)	η_10_ (mV)	TOF/s^−1^	References
P-Ru/C	P	0.03	31	N/A	[31]
S-RuP@NPSC	N, P, S	0.36	92	N/A	[36]
Ru@CN	N	0.25	32	N/A	[29]
Ru NCs/BNG	B, N	0.71	14	N/A	[33]
Ru/S-rGO	S	0.5	3	1.86 @ 50 mV	[28]
RuNi/CQDs	Ni	0.42	13	5.03 @ 100 mV	[32]
2DPC-RuMo	Mo	0.32	18	3.57 @ 50 mV	[47]
RuCo@NC	Co	0.28	28	N/A	[41]
3D RuCu NCs	Cu	1.33	18	N/A	[49]

## Support Engineering

As an important component of the catalyst, the catalytic enhancement mechanism of the support must be understood to reasonably design the catalyst. The support can effectively disperse the metal active sites, increase the specific surface area of the catalyst, expose the active sites, and change the number and combination of the active sites of the catalyst. The combination of support and metal active components often leads to new interface phenomena, such as the formation of chemical bonds between metals and carriers and the charge transfer between metals and carriers, called metal–support interactions (MSI) [50]. In the preparation and use of catalysts, regulating the MSI significantly impacts the performance and characteristics of catalysts. Therefore, the design of the catalyst support is also vital in improving the catalytic activity of Ru-based HER. In recent years, different morphologies of carbon and metal-oxide supports have been studied extensively.

### Carbon Supports

Owing to their low cost and excellent conductivity, carbon materials are widely used as supports for various catalysts. After the structure and composition control, the catalytic performance can be greatly enhanced. For example, by designing porous structures [51], increasing the specific surface area of catalysts to fully expose the metal active sites, and through heteroatom doping [52], the anchor points of the active components can be increased. Through various efforts, we aim to protect the aggregation of active sites in the reaction and the activity in various reaction media, thereby enhancing the catalyst’s activity and stability.

Recently, Ru-based graphite/graphene composites have shown excellent performance in the HER owing to their excellent electronic transport properties and unique geometric structure [53]. Wang et al. [54] reported the formation of Ru NPs encapsulated in nitrogen-doped graphite carbon foam (Ru-NGC) by pyrolysis. A small amount of Ru NPs was surrounded by several layers of nitrogen-doped graphitized carbon shells with an interlayer distance of 0.34 nm. The catalyst exhibited good HER performance and stability owing to the uniform distribution of Ru NPs and the protection of the graphite layer. Ru-NGC shows a similar overpotential (25 vs. 29 mV) and Tafel slope (31 vs. 29 mV dec^−1^) to Pt/C at a current density of 10 mA cm^−2^ in 0.5 M H_2_SO_4_. Graphene support derived from graphite is also an excellent choice as catalyst support. Baek et al. [55] reported that highly dispersed Ru particles (2 nm) were loaded on graphene (Ru@GnP), which exhibited outstanding HER activity in acidic and alkaline media. The synthesis of Ru@GnP was divided into two steps: the synthesis of the support and loading reduction in the metal. Graphite was ball-milled in the presence of dry ice to prepare the edge-carboxylic-acid-functionalized graphene nanoplatelets (CGnP). Subsequently, the Ru ions were combined with CGnP, and Ru@GnP was synthesized after the reduction and annealing treatment. In this process, the rich carboxylic acid groups on the support were coordinated with Ru ions, enhancing the combination of Ru ions and the support, thus improving the loading of Ru. The as-prepared Ru@GnP showed small overpotential at 10 mA cm^−2^ (13 mV in 0.5 M H_2_SO_4_ and 22 mV in 1 M KOH) and low Tafel slopes (30 mV dec^−1^ in 0.5 M H_2_SO_4_ and 28 mV dec^−1^ in 1 M KOH). Moreover, after 10,000 cycles of long-term durability testing, it still maintains its high dispersion morphology due to the high surface area (403.04 m^2^ g^−1^) of the carrier that prevents the aggregation of Ru particles in the reaction. Large quantities of highly dispersed Ru-based catalysts prepared by mechanochemical-assisted synthesis have practical applications.

**Fig. 3:**
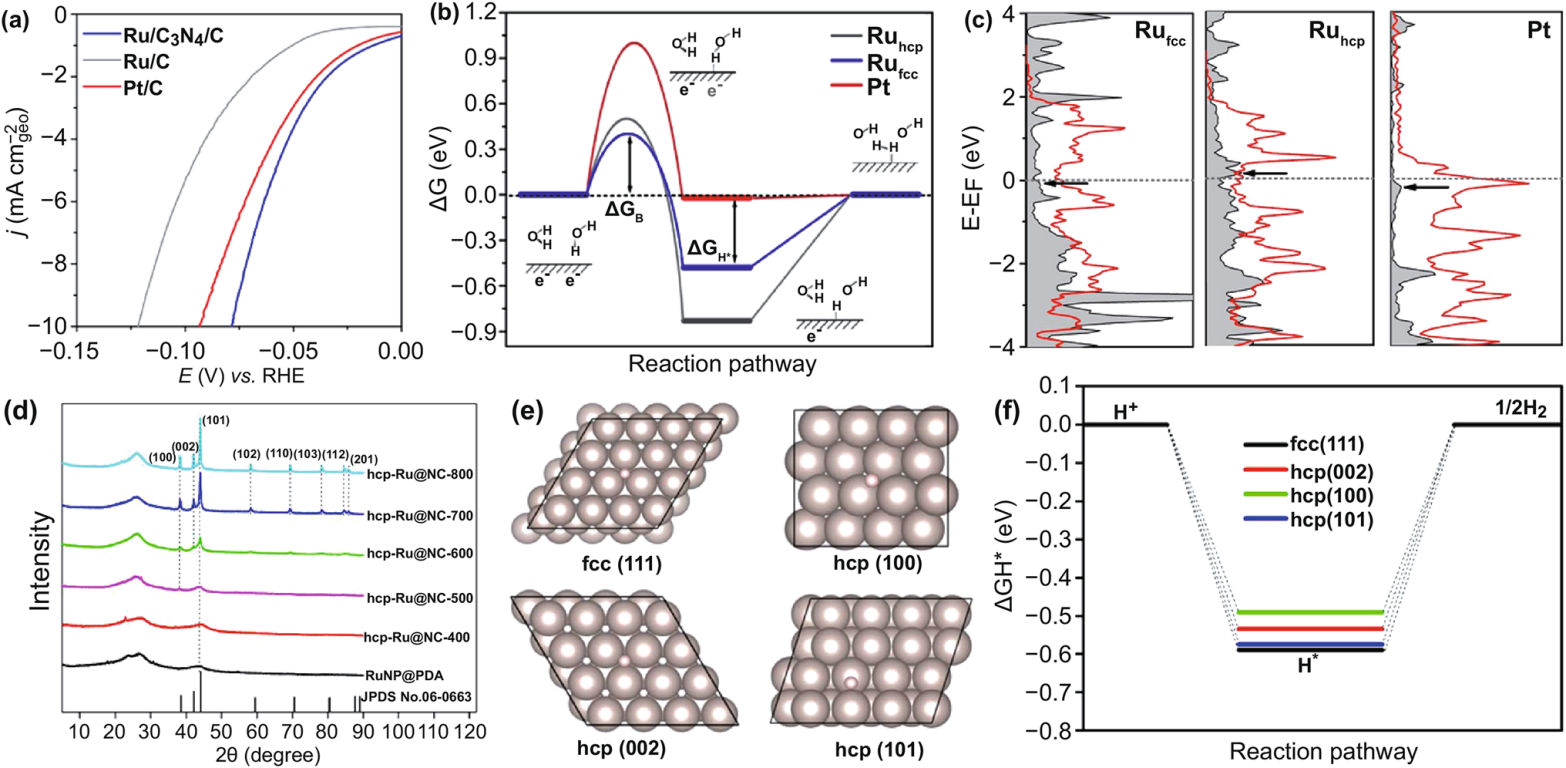
**a–c** HER polarization curves, Gibbs free energy diagram of HER on different metal surfaces and local density of states projected for the adsorbed H atom (H-DOS, dark shaded area) on three metal surfaces [35], with permission from the American Chemical Society. **d–f** XRD pattern of RuNP@PDA and hcp-Ru@NC annealed at various temperatures ranged from 400 to 800 ℃, H adsorption models and ΔG_H*_ on different Ru surfaces, respectively [61], with permission from the American Chemical Society

Nitrogen-doped carbon carriers are currently one of the commonly used catalyst carriers. As the atomic size of nitrogen is similar to that of carbon, the lattice disorder of the material can be reduced by doping with nitrogen [56], and nitrogen activates adjacent carbon atoms to increase the density of active centers [57]. The prepared materials have excellent catalytic activity owing to the electronic regulation of nitrogen [58–60]. Ru/C catalyst only exhibits a sole hexagonal close packed (hcp) phase, while Qiao et al. [35] found that C_3_N_4_ can induce Ru to form a new face-centered cubic (*fcc*) crystallographic structure. It was indicated that the hydrogen evolution TOF of this structure under alkaline condition was 2.5 times that of Pt/C. This study provides a deep understanding of the origin of HER activity in different Ru crystal structures. The experiments showed that moderate nitrogen doping could increase the conductivity of the carrier, effectively regulate the electronic structure of Ru, and improve the HER activity (Fig. 3a). However, excessive nitrogen content would destroy the conjugated structure of the carbon skeleton and reduce the conductivity [29]. The calculation shows that the lower d-band center of Ru, the weaker H adsorption, such as Pt, while for Ru_hcp_, the higher d-band position is unsatisfactory for H desorption, which reduces the rate of Heyrovsky step and results in a poor HER activity for Ru/C. In contrast, by introducing the face-centered cubic phase (Ru_fcc_), the d-band center of Ru_fcc_ was found to be lower than that of Ru_hcp_, thus optimizing the H desorption, and the water dissociation free energy barrier (ΔG_B_) and ΔG_H*_ of Ru_fcc_ were more inclined to zero as compared with Ru_hcp_ (Fig. 3b, c), and the HER activity of the catalyst was improved. In addition, according to Liu et al. [61], the HER activities of different Ru crystalline surfaces were found to be in the order of hcp (100) > hcp (002) > hcp (101) > fcc (111), and with increasing the heat treatment temperature, hcp (100) and (002) were gradually exposed and crystallinity increased, enhanced HER activity was obtained (Fig. 3d–f). Therefore, great effort still needs to be invested in revealing the origin of the intrinsic activity on different crystalline planes of Ru to develop the advanced Ru-based catalysts.

**Fig. 4:**
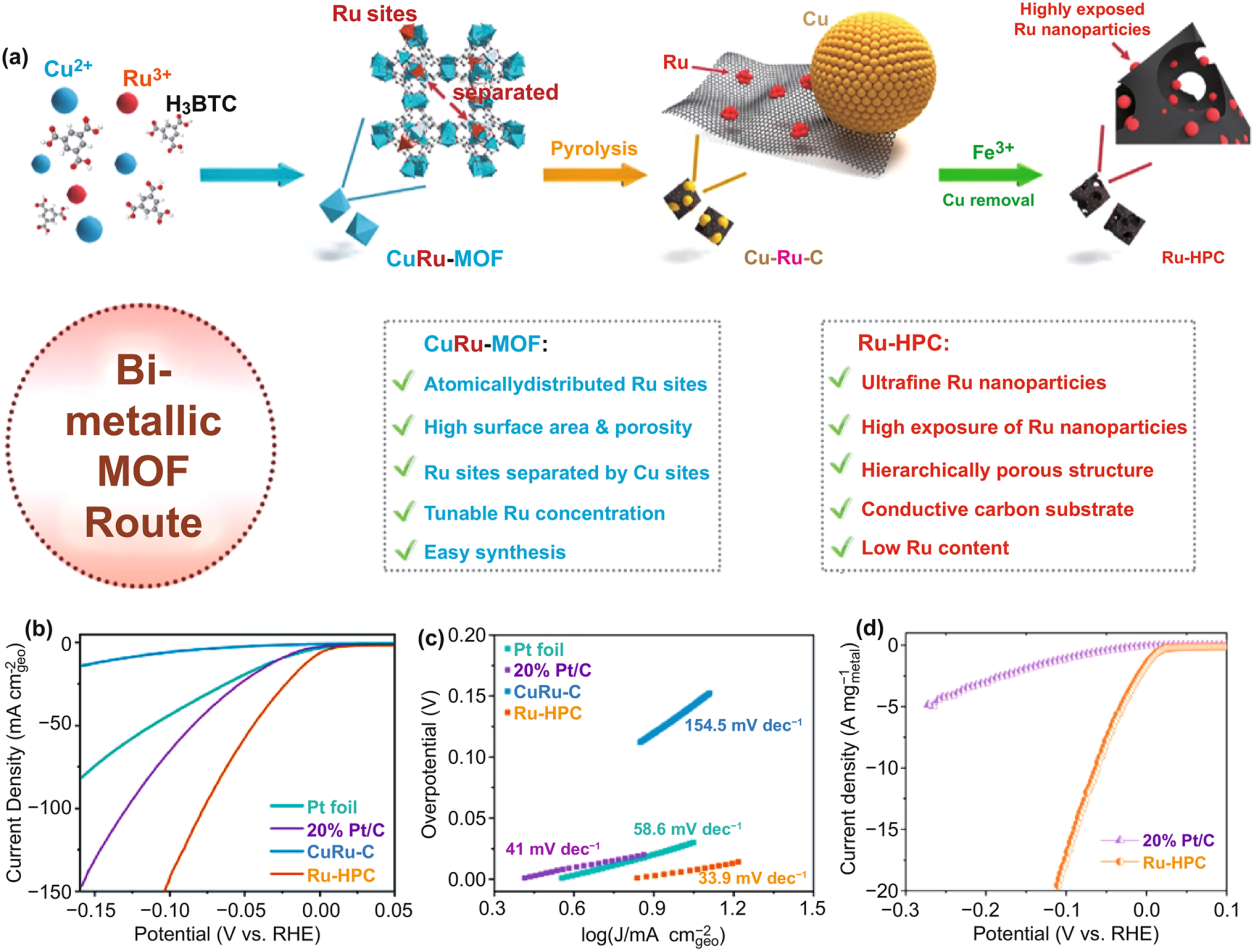
**a** Schematic illustration of the synthetic strategy of Ru-HPC. **b–d** Polarization curve, Tafel slope and mass activity curve of Ru-HPC at 1 M KOH [63], with permission from the Elsevier Ltd

Metal–organic frameworks (MOFs) have porous structure generated by the highly ordered arrangement of organic connectors and metal nodes, making them ideal supports for loading catalysts. Porous carbon materials derived by heat treatment have unique performance characteristics and advantages in the field of catalysis [62]. Zou et al. [63] loaded Ru in Cu-MOF as the precursor, followed by pyrolysis and removal of Cu to prepare Ru-decorated hierarchically porous carbon (Ru-HPC) for the HER (Fig. 4). The presence of Cu sites prevented the aggregation of Ru during pyrolysis. After Cu sites removal, many meso-/macropores were generated in Ru-HPC, fully exposing the active sites of Ru and effectively preventing the aggregation of Ru in the reaction. Owing to its unique structure, Ru-HPC showed an extremely high electrochemical surface area and high active sites density. The mass activity of Ru-HPC was 19 times that of Pt/C under alkaline condition. This material synthesis method provides ideas for the preparation of high-performance metal–carbon hybrid electrocatalysts with abundant exposed active sites. Additionally, 2D Ru-based catalyst (Ru-MIL-53(NiFe)) [64] and single-atom Ru-based catalyst (Ru-Co NPs@N–C) [65] have also been reported using MOF as the precursor carrier.

### Metal-oxide Supports

The application of Ru-based catalysts in different environments can be increased by developing different carriers. In view of the good OER performance of metal oxides, the development of bifunctional catalysts with OER and HER activities based on metal oxides has gathered attention [4]. Metal oxides have attracted great attention in the field of catalysis due to their diverse composition/structure, low cost, high abundance, easy synthesis and environmental friendliness [66–68]. Compared with carbon supports, metal oxide supports tend to have stronger interaction with metal catalysts at the contact interface, thus bringing stronger stability to the catalyst. However, metal oxides generally suffer from low electrical conductivity compared with the carbon supports, limiting its application for HER [69–71]. Therefore, the issue of insufficient conductivity of metal oxide supports should be primarily resolved before the application of metal oxides as the supports to improve the performance and stability of Ru-based catalysts for HER.

**Fig. 5:**
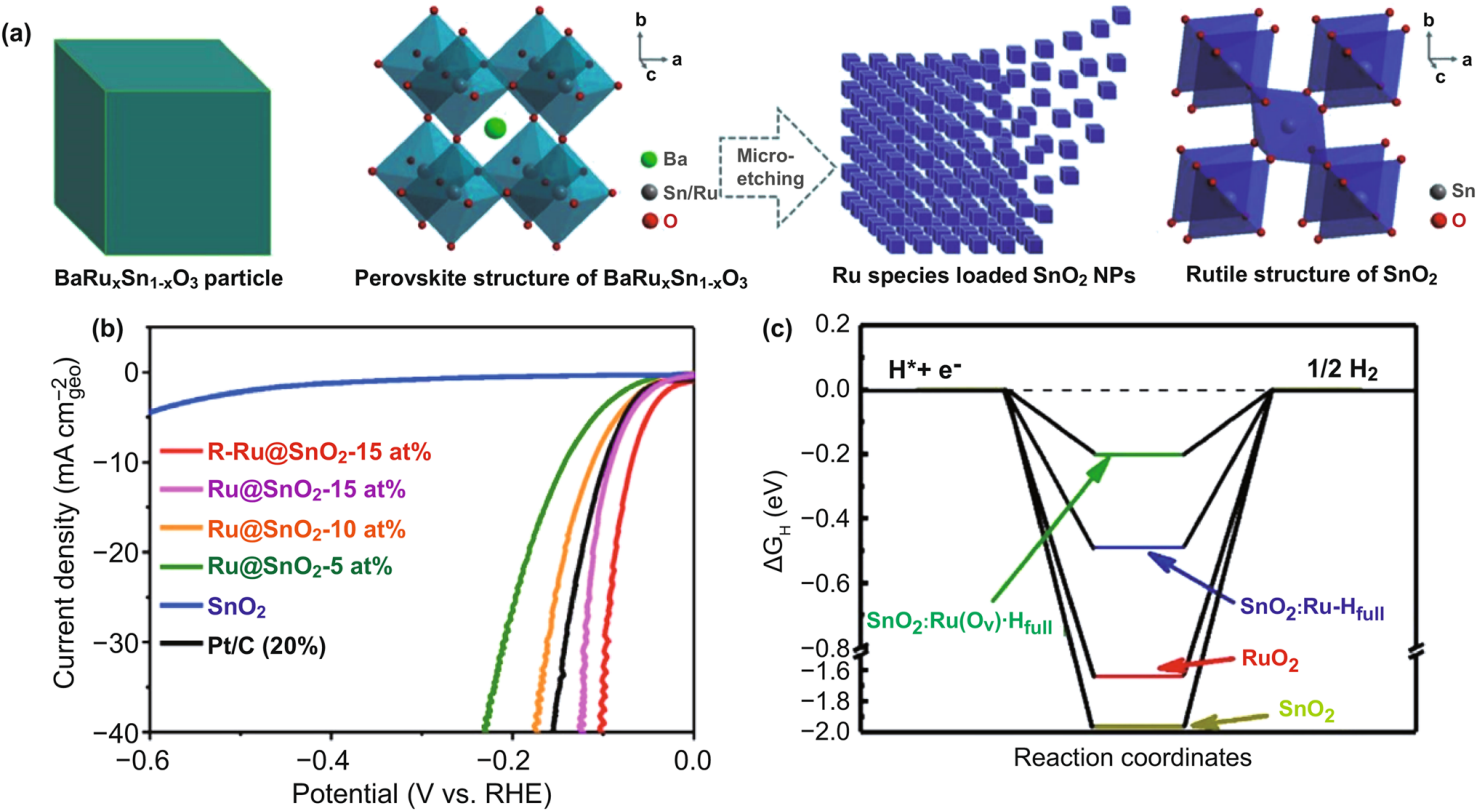
**a** Schematic illustration of the synthetic strategy of Ru@SnO_2_. **b** Polarization curve of Ru@SnO_2_ at 0.1 M KOH. **c** HER energy diagram [73], with permission from the Cell Press

Recently, Ru-loaded metal oxidation (CeO_2_ [72], SnO [73], MoO_2_ [74], TiO [75], NiO [76], CoO [77], and RuO [78]) for the HER has been extensively studied, and high HER activity and durability have been reported because of the strong MSI between Ru metal and its oxides. Akbayrak et al. [79] reduced Ru^3+^ ions on CeO_2_ to Ru NPs using an aqueous solution of NaBH_4_ to form Ru^0^/CeO_2_. Owing to the transition from Ce^4+^ to Ce^3+^ in CeO_2_, there are abundant oxygen defects in the material. The existence of these defects is beneficial to the transport and conduction of electrons, which solves the inherent shortcomings of the poor conductivity of most metal oxides while also improving the catalytic activity. The prepared Ru^0^/CeO_2_ has a low overpotential of 47 mV (10 mA cm^−2^ in 0.5 M H_2_SO_4_) and excellent stability after 10,000 cycles of the CV durability test. The strategy of oxygen vacancy enhancement of the HER activity was also confirmed for other metal-oxide supports. Ling et al. [77] prepared a Ru/CoO hybrid electrocatalyst that also enhanced the HER activity using oxygen vacancies on the CoO carrier. Huang et al. [73] reported a subnano-Ru species anchored on nano-SnO_2_ (Ru@SnO_2_). Subnano-Ru anchored on nano-SnO_2_ was prepared using a space-confined and lattice-confined effect-assisted micro-etching method, and the Ru@SnO_2_ displayed high activity for the alkaline HER with a low overpotential and small Tafel slope (Fig. 5). Similarly, enhancing the MSI has also been confirmed as a method for improving the stability of catalysts. Chen et al. [80] reported the preparation of Ru-MoO_2_ nanocomposites through a facile in situ carburization of a Ru modified Mo-based metal–organic framework. The Ru-MoO_2_ showed low overpotential and superior stability at current density of 10 mA cm^−2^ (29 mV in 1 M KOH and 55 mV in 0.5 M H_2_SO_4_). The experiments show that the excellent stability of Ru-MoO_2_ arises from the strong interaction force between Ru and Mo, which protects the agglomeration of Ru in the reaction. Additionally, through DFT calculations, the Δ*G*_H*_ value of MoO_2_ and Ru were positive and negative, respectively. Therefore, through the combination of the two materials, the Δ*G*_H*_ value of Ru-MoO_2_ was approximately 0 eV. Thus, the HER overpotential was reduced by optimizing the adsorption and desorption capacities of H on the material. The performances of Ru-based HER catalysts with different supports in recent years are presented in Table 3.

**Table 3:** HER performance under different supports at 1.0 M KOH

Catalysts	Carriers	Loading (mg cm^−2^)	η_10_ (mV)	TOF/s^−1^	References
Ru@GnP	Graphene	0.25	22	N/A	[55]
Ru/C3N4/C	C_3_N_4_	0.20	79	4.2 @ 100 mV	[35]
Ru/triNC	C_3_N_3_	0.51	2	1.26 @ 40 mV	[101]
Ru@Co-SAs/N–C	MOFs (ZIF)	0.29	7	N/A	[65]
Ru/CoO	CoO	0.20	55	N/A	[77]
Ru-MoO_2_	MoO_2_	0.28	29	N/A	[80]
Ru^0^/CeO_2_	CeO_2_	0.19	47 (0.5 M H_2_SO_4_)	0.8 @ 27 mV	[79]
Ru@SnO_2_	SnO_2_	0.25	68 (0.1 M KOH)	2.7 @ 100 mV	[73]

## Low-Dimensional Nanostructures Design

Due to the non-directional nature of metal bonds, metal atoms tend to form three-dimensional tightly packed nanoparticles [81, 82], leading to the low atom utilization and poor catalytic performance of zero-dimensional (0D) metal materials. Additionally, the detachment, migration, and sintering phenomena of the 0D materials further result in inferior stability. One-dimensional (1D) (nanotubes [83, 84], nanorods [85]) and two-dimensional (2D) (nanosheets [86], nanoplates [87]) metal materials have been widely studied and applied owing to their higher electron transfer rate and anisotropic properties compared to those of 0D materials, which can effectively improve the activity and durability of Ru-based catalysts.

### 1D Ru-Based Catalyst

**Fig. 6:**
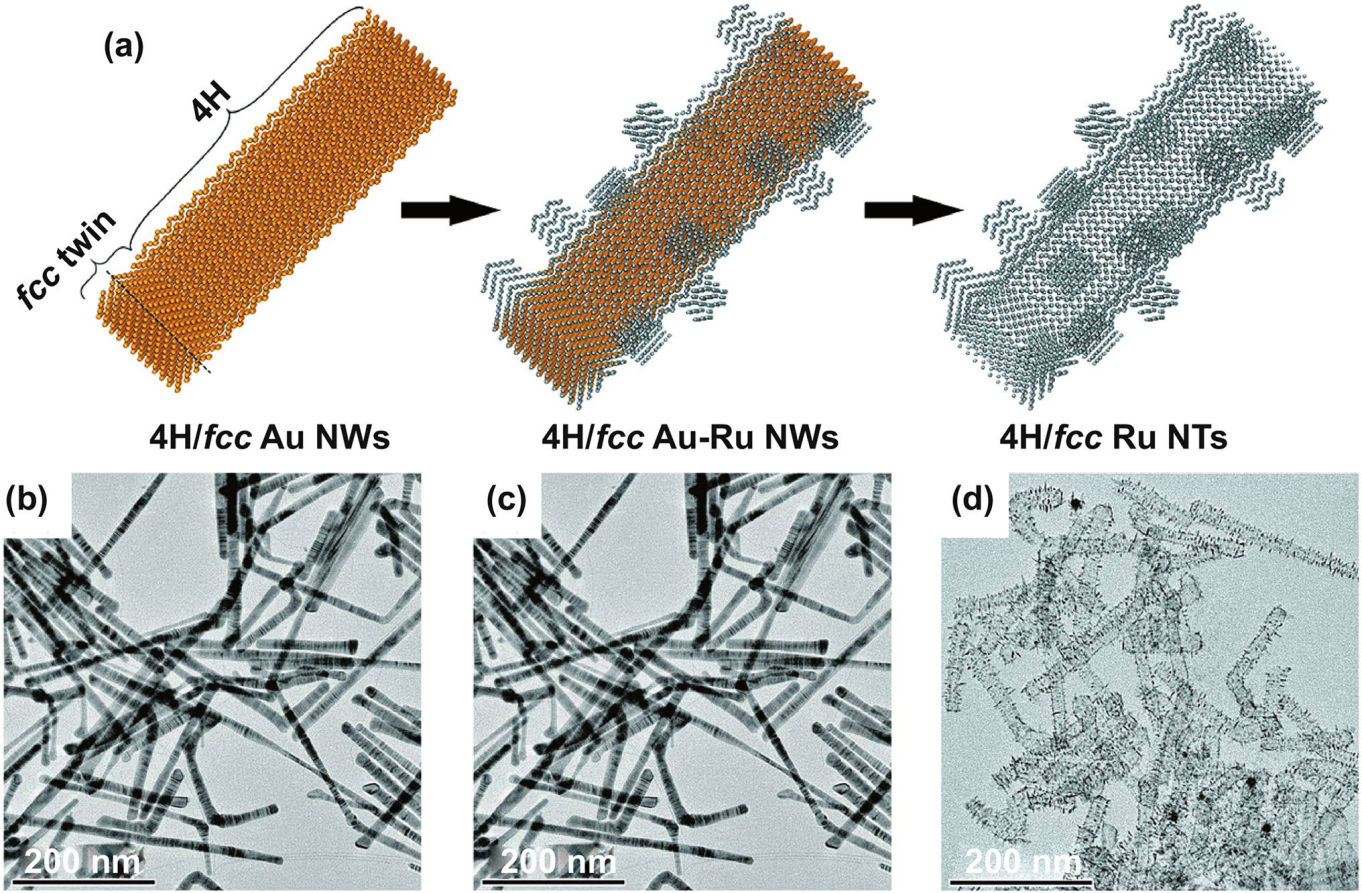
**a** Schematic illustration of the synthesis of hierarchical 4H/fcc Ru NTs. **b–d** Low-magnification TEM images of 4H/fcc Au NWs, 4H/fcc Au-Ru NWs, and 4H/fcc Ru NTs, respectively [83], with permission from the Wiley–VCH Verlag GmbH & Co. KGaA, Weinheim

The synthesized 1D nanomaterial usually has abundant defect sites and lattice distortion due to the high surface energy of metal atoms; unsaturated electronic coordination sites such as this have been confirmed to exhibit a high HER activity. Gu et al. [83] prepared hierarchical 4H/face-centered cubic (*fcc*) Ru nanotubes (NTs) using the hard template method, wherein 4H/fcc Au nanowires (NWs) served as sacrificial templates that were then etched by copper ions (Cu^2+^) in dimethylformamide (Fig. 6). The wall thickness of Ru NTs ranged from 5 to 9 atomic layers. In an alkaline medium, Ru NTs showed better HER performance than commercial Pt/C, and after 10,000 cycles of the stability test, Ru NTs maintained their 1D tubular structure. Thus, the 1D structure of Ru-based catalysts provides a larger surface area and active sites for the catalytic reaction, and the rich defects and lattice distortion caused by Ru NTs greatly improve the electrocatalytic hydrogen evolution performance. Based on the defect effect and lattice distortion, Ru alloy 1D materials also showed excellent HER activity. Huang et al. [85] developed 1D amorphous RuTe_2_ porous nanorods (PNRs). The introduction of Te effectively eliminated the crystal-field-splitting effect at the Ru sites, stabilized the distorted strain, and increased the electronic activity near the Fermi level. Within this trend, the locally distorted Ru-Te lattice increased the homogeneity for an efficient inter-d-orbital electron transferability among Ru sites while also improving the catalytic activity.

### 2D Ru-Based Catalyst

**Fig. 7:**
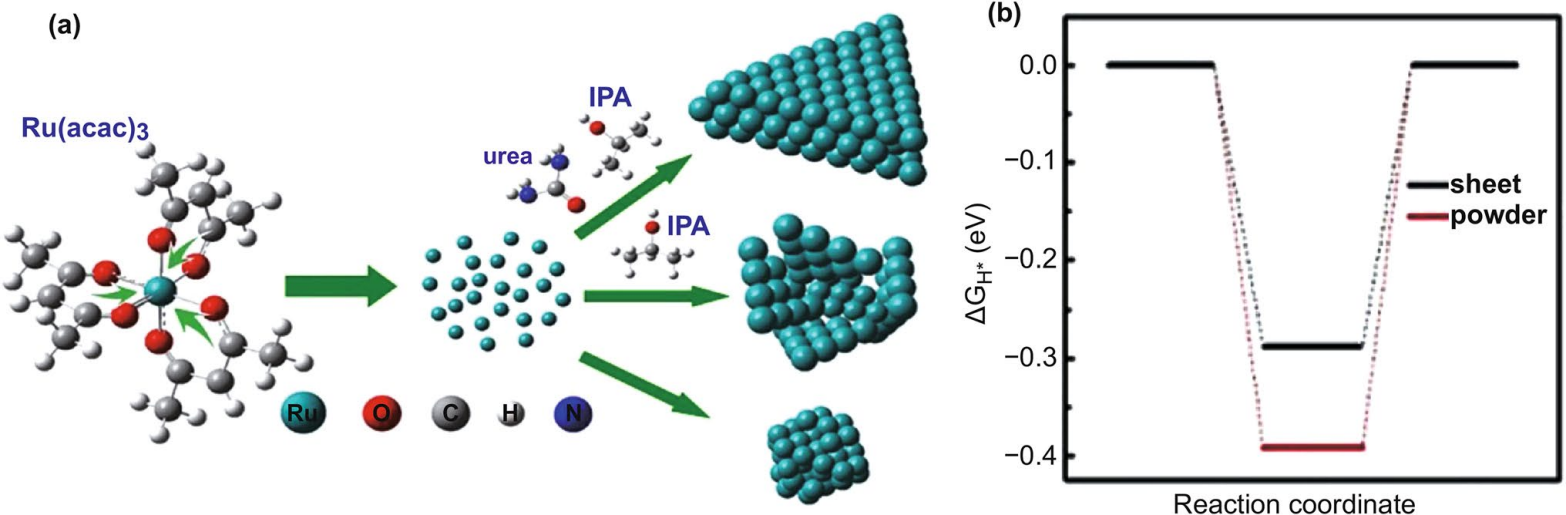
**a** Schematic illustration of the growing process of Ru nanosheets. **b **ΔG_H*_ value of Ru nanosheet and powder [88], with permission from the American Chemical Society

Ultrathin 2D nanosheets provide a large surface area and 2D permeable channels for ion adsorption and transport [88]. 2D Ru metal nanosheets also show great potential for the HER. Peng et al*.* [86] used isopropanol (IPA) and urea to control the reduced through the self-decomposition of Ru acetylacetonate (Ru(acac)_3_) to form ultrathin 2D nanosheets with a thickness of only 0.21 nm (Fig. 7). In the system, IPA helps anisotropic growth into sheet-like structures, and urea prevents their accumulation and contributes to the growth of larger sheets. Ultrathin Ru nanosheets exhibit the HER performance similar to that of commercial Pt/C, and DFT calculations shows that the Δ*G*_H*_ of nanosheets (− 0.289 eV) is closer to 0 than the Ru power ( − 0.392 eV). This shows the potential application of Ru nanosheets in the HER. Based on the development of Ru nanosheets, the structural optimization and composition studies of Ru nanosheets are particularly important for further improving their HER catalytic performance. As previously discussed, the introduction of impurity elements can optimize the electronic structure of Ru to effectively improve the HER performance, which is also effective for 2D Ru nanosheets. A 2D HER/OER bifunctional RuCu snowflake-like nanosheets catalyst was designed by Huang et al. [89], which only required cell voltages of 1.49, 1.55, 1.49, and 1.50 V (in 1 M KOH, 0.1 M KOH, 0.5 M H_2_SO_4_, and 0.05 M H_2_SO_4_, respectively) to drive water splitting at a current density of 10 mA cm^−2^. Owing to the channel-rich structure of the RuCu NSs, their electron transferability was greatly improved, and the electron structure of the oxidation and reduction in water splitting was optimized. The excellent HER catalytic performance of low-dimensional Ru-based nanomaterials is encouraging; however, the design of novel synthesis methods to effectively control their 1D/2D morphology still requires continuous research. The properties of Ru-based HER catalysts with low-dimensional nanostructures are presented in Table 4.

**Table 4:** HER performance in 1D/2D structure at 1.0 M KOH

Catalysts	1D/2D structure	Loading (mg cm^−2^)	η_10_ (mV)	TOF/s^−1^	References
4H/fcc Ru NTs	1D Nanotube	0.034	23	0.22 @ 30 mV	[83]
RuTe_2_ PNRs	1D Nanorods	0.20	36	N/A	[85]
RuCu NSs	2D nanosheets	N/A	20	N/A	[89]
Ru nanosheets	2D nanosheets	0.10	20 (0.5 M H_2_SO_4_)	N/A	[86]

## Maximum Utilization: Single-atom Ru-based Catalysis

As the concept of single-atom catalysis (SACs) was proposed by Zhang et al. [90] in 2011, SACs have become important materials in various catalytic fields. The isolated active centers often have strong interactions with coordination species or large amounts of electron transfer, making SACs exhibit properties different from those of the nanocatalysts. SACs may be the best solution for Ru-based catalysts because of the maximum utilization of Ru atoms and the exposure of active centers. Presently, Ru SACs with different loadings have been prepared on various carriers, such as phosphors [91, 92], MOF [93], Graphdiyne [94], MXene [95, 96], and nitrogen-doped carbon [97].

**Fig. 8:**
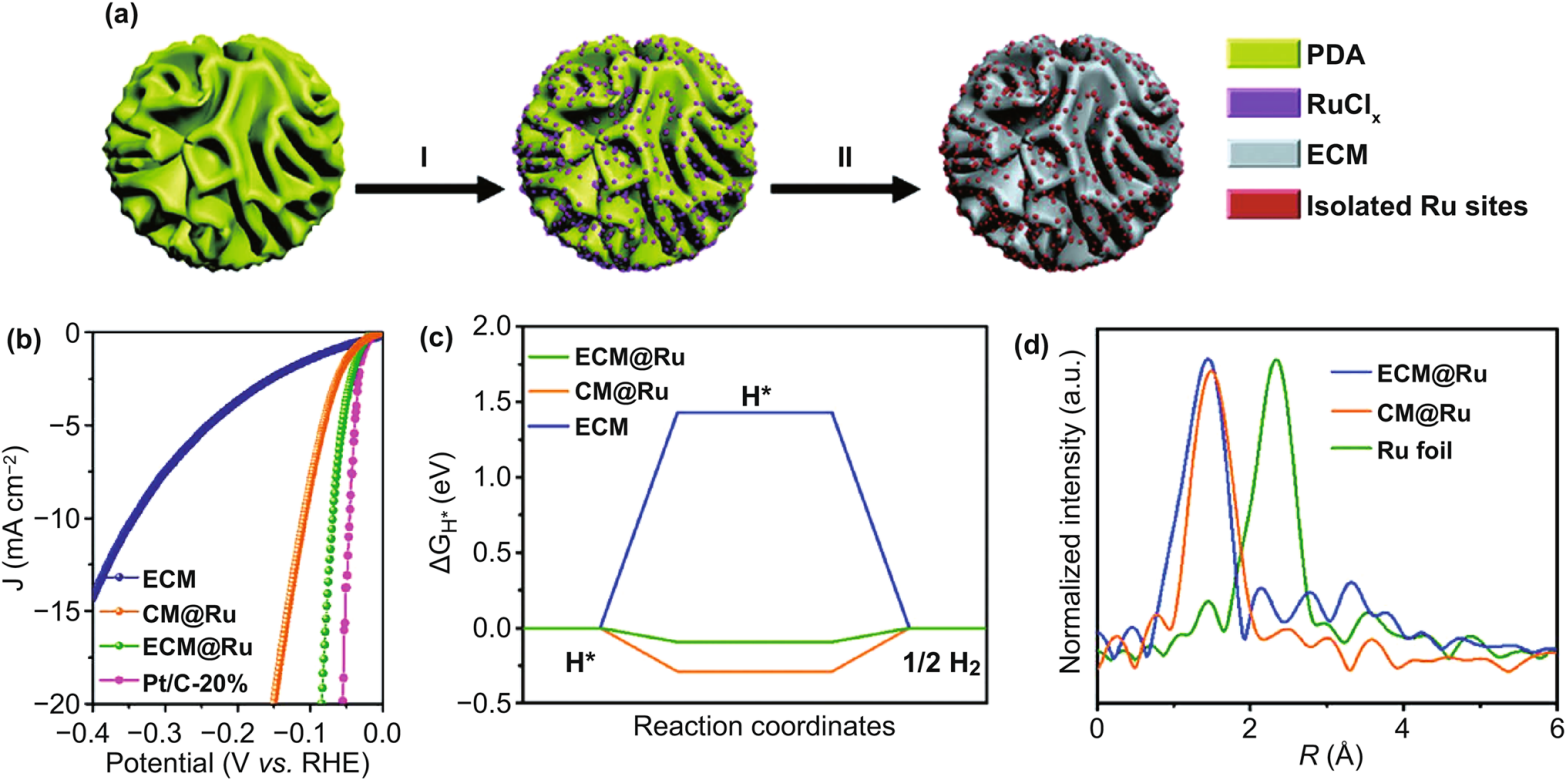
**a** Schematic illustration of the synthetic procedure of ECM@Ru. **b** Polarization curve of ECM@Ru at 0.5 M H_2_SO_4_. **c** Calculated ΔG_H*_ on ECM@Ru, CM@Ru, and ECM. **d** Fourier-transformed magnitudes of the experimental Ru K-edge EXAFS spectra [97], with permission from the Wiley–VCH Verlag GmbH & Co. KGaA, Weinheim

The metal–nitrogen (M–N) structure is considered a stable structure favorable for single-atom formation [98]. Kim et al. [99] reported SACs with a Ru–N structure and confirmed that N is the anchor coordination site of a single Ru atom by X-ray absorption fine structure (EXAFS). Moreover, through the coordination of N, the binding ability of Ru to the HER intermediate H was optimized, thereby enhancing the HER activity of Ru-SACs. Based on the study on single Ru atoms, the modification of the active sites can also enhance the catalytic activity. Compared with the surface of carbon materials, the density of electronic states at the edge of carbon materials is denser with higher activity [100]. Therefore, loading Ru atoms on the edge of the carbon matrix is another way to reinforce the catalyst activity. Lou et al. [97] developed SACs for the modification of isolated Ru sites with a precise configuration into an edge-rich carbon matrix (ECM@Ru). Polydopamine (PDA) was used as the precursor of carbon carriers to produce edge-rich carbon carriers and provide N-anchored sites for the Ru atoms (Fig. 8). Subsequently, the Ru atoms were fused into the edge of the carbon matrix support via high-temperature pyrolysis. In acidic medium, the mass activities of the prepared ECM@Ru at overpotential of 50 and 100 mV were 6.4 and 9.6 times those of commercial Pt/C, respectively. Additionally, ECM@Ru also exhibited HER performance similar to that of commercial Pt/C under alkaline conditions. Aggregation of active sites at the edge of the support resulted in the electron enrichment around the Ru sites, which enhanced the local electric field and accelerated the catalytic kinetics.

**Fig. 9:**
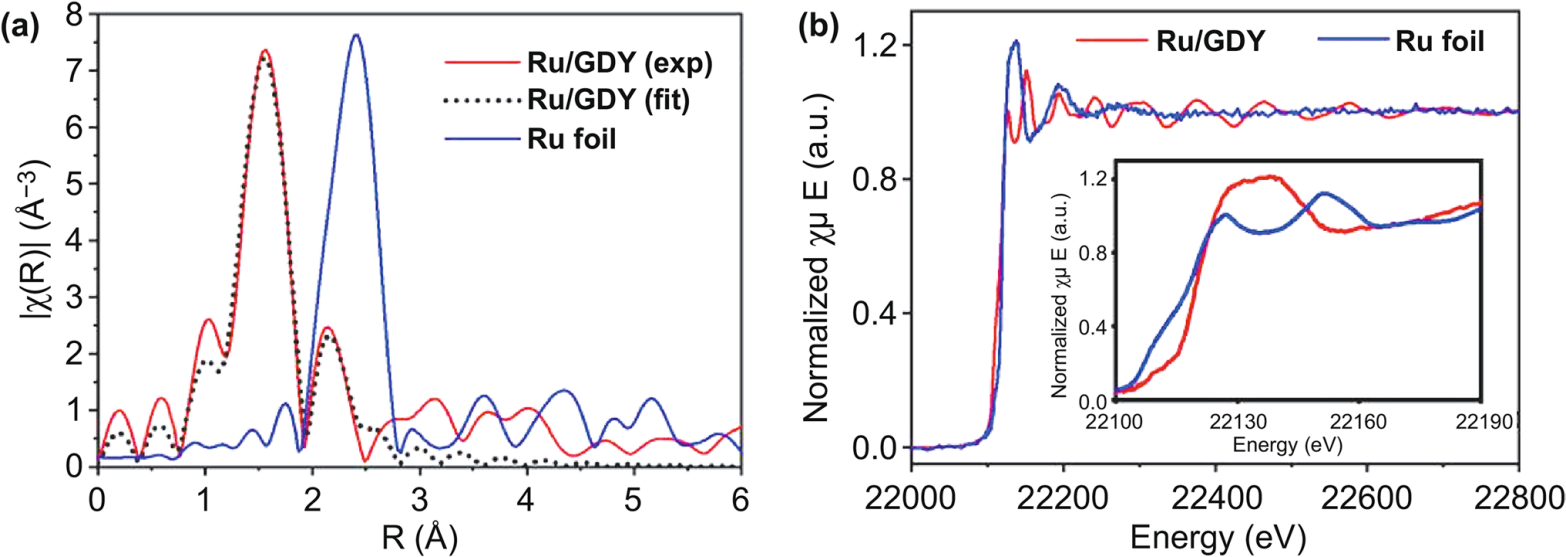
**a** Experimental and fitted EXAFS spectra of Ru/GDY and Ru foil. **b** K-edge XANES spectra of Ru/GDY and Ru foil [94], with permission from the Elsevier Ltd

Given the mature industrial system and the large-scale commercial application of carbon-based catalysts, the activity and stability mechanism of SACs is easy to understand by simplifying the catalyst components. Li et al. [94] prepared Ru/GDY by loading single Ru atoms in graphdiyne (GDY). DFT calculations and EXAFS analyses indicated strong *p-d* coupling between the Ru and adjacent C atoms that formed a Ru–C bond to stabilize the Ru single-atom (Fig. 9). In acidic medium, the HER overpotential of the prepared Ru/GDY at a current density of 10 mA cm^−2^ is only 44 mV and has excellent long-term stability. This work reveals the bonding relationship between Ru and C and provides a valuable reference value for the study of Ru/C systems. The visual morphology and composition of single atoms can be characterized by high-resolution transmission electron microscopy (HRTEM) and synchrotron radiation spectroscopy based on the advanced characterization methods. The future challenges for Ru single-atom catalysts are effectively using these advanced research methods, deepening the understanding of the intrinsic origin of catalytic activity at the atomic level of catalysts, optimizing the preparation process of SACs, and rationally designing highly efficient HER catalysts. The properties of the monoatomic-ruthenium-based HER catalysts are presented in Table 5.

**Table 5:** HER performance doped with different single atom coordination environments at 1.0 M KOH

Catalysts	Coordination environment	Loading (mg cm^−2^)	η_10_ (mV)	TOF/s^−1^	References
RuAu SAAs	Ru-Au	0.28	24	2.18 @ 50 mV	[102]
ECM@Ru	Ru–N	N/A	63 (0.5 M H_2_SO_4_)	N/A	[97]
Ru_1_CoP/CDs	Ru–P	0.42	51	N/A	[91]
Ru_SA_-N-S-Ti_3_C_2_T_x_	Ru–N, Ru–S	1.00	76 (0.5 M H_2_SO_4_)	0.52 @ 100 mV	[95]
Ru SAs–Ni_2_P NPs	Ru–P, Ru-Ni	0.50	57	1 @ 57 mV	[92]
NiRu_0.13_-BDC	Ru–O	2.50	34	0.0091 @ 100 mV	[93]
Ru@Co-SAs/N–C	Ru–N	0.29	7	N/A	[65]
Ru-SA/Ti_3_C_2_T_x_	Ru–O	0.61	70 (0.1 M HClO_4_)	2.67 @ 100 mV	[96]
Ru/GDY	Ru–C	0.48	44 (0.5 M H_2_SO_4_)	8.45 @ 100 mV	[94]

## Conclusion and Perspectives

H_2_ has broad application prospects in the future, and it is vital in alleviating the energy crisis, greenhouse effects, and air pollution. However, a new type of energy, from its research and development to application stages, requires considerable effort to be practically applicable. Based on this, a great process has been achieved in highly effective HER electrocatalysts for H_2_ production. In this review, based on HER mechanisms during the electrochemical water-splitting process, four strategies to improve the performance of Ru-based electrocatalysts are discussed.

Although Ru-based catalysts are seemingly ideal substitutes for currently used Pt-based catalysts, the application and development of Ru-based catalysts are still in their infancy because of the limited research results. Presently, research on Ru-based catalysts is still at the laboratory level, while industrial applications require maximum results for minimum expenditure. Thus, the development of facile and cost-effective methods to synthesize effective Ru-based catalysts is still highly required for their practical production and application. Additionally, the majority of the research data (activity and durability) are still calculated using three-electrode systems. However, for practical applications, the catalysts are operated in more complex conditions, considering factors such as gas diffusion, mass and electron transfer resistance, and accessible active sites. Therefore, it is particularly important to develop a representative test system for practical applications. Furthermore, acid or alkaline electrolytes are presently the general choice of catalyst application systems, but such media will bring challenges to the service lives of electrolysis devices. Thus, designing catalysts that can also be effective in neutral environments is necessary. Additionally, by applying resource-rich solar, wind, and tidal energy to the in situ electrochemical seawater splitting, and thus converting these idle energies for H_2_ production, the applicability of electrolytic water can be expanded in the future. However, both the corrosive condition and chlorine evolution reaction cast a shadow on the highly effective and durable catalysts. Lastly, using current advanced characterization methods, the composition of the catalysts and mechanisms of the HER process can be explained on the atomic level. To further understand the change of state in the reaction process and reveal the real active center and reaction intermediates, in situ characterization methods such as in situ XRD, TEM, and Raman spectroscopy are needed. In summary, Ru-based catalysts have broad application prospects, and hydrogen will gradually replace fossil-based energy and become a vital component of future energy structures through continuous in-depth research.
